# JFD, a Novel Natural Inhibitor of Keap1 Alkylation, Suppresses Intracellular *Mycobacterium Tuberculosis* Growth through Keap1/Nrf2/SOD2-Mediated ROS Accumulation

**DOI:** 10.1155/2023/6726654

**Published:** 2023-02-10

**Authors:** Haoqiang Wan, Yi Cai, Lingyun Xiao, Yunzhi Ling, Lanlan Ge, Siwei Mo, Qiujie Xie, Shusong Peng, Boping Zhou, Xiaobin Zeng, Xinchun Chen

**Affiliations:** ^1^Center Lab of Longhua Branch and Department of Infectious Disease, Shenzhen People's Hospital (The Second Clinical Medical College, Jinan University; The First Affiliated Hospital, Southern University of Science and Technology), Shenzhen, 518020 Guangdong Province, China; ^2^Department of Pathology (Longhua Branch), Shenzhen People's Hospital, 2nd Clinical Medical College of Jinan University, Shenzhen, 518020 Guangdong Province, China; ^3^Guangdong Key Laboratory of Regional Immunity and Diseases, Department of Pathogen Biology, Shenzhen University School of Medicine, Shenzhen, 518120 Guangdong Province, China

## Abstract

It is an effective strategy to treat tuberculosis by enhancing reactive oxygen species- (ROS-) mediated killing of *Mycobacterium tuberculosis* in macrophages, but there are no current therapeutic agents targeting this pathway. *Honeysuckle* has been used as the traditional medicine for tuberculosis treatment for 1500 years. Japoflavone D (JFD) is a novel biflavonoid isolated from *Honeysuckle* promoting ROS accumulation by Nrf2 pathway in hepatocarcinoma cells. However, its activity to kill *M. tuberculosis* in macrophages and molecular mechanism has not been reported. Our results showed that JFD enhances the *M. tuberculosis* elimination by boosting ROS levels in THP-1 cells. Moreover, the massive ROS accumulation activates p38 to induce apoptosis. Notably, the mechanism revealed that JFD suppresses the nuclear transport of Nrf2, thereby inhibiting SOD2 transcription, leading to a large ROS accumulation. Further studies showed that JFD disrupts the Keap1 alkylation at specific residues Cys14, Cys257, and Cys319, which is crucial for Nrf2 activation, thereby interrupts the nuclear transport of Nrf2. In pharmacokinetic study, JFD can stay as the prototype for 24 h in mice and can be excreted in feces without any toxicity. Our data reveal for the first time that a novel biflavonoid JFD as a potent inhibitor of Keap1 alkylation can suppress the nuclear transport of Nrf2. And it is the first research of the inhibitor of Keap1 alkylation. Furthermore, JFD robustly promotes *M. tuberculosis* elimination from macrophages by inhibiting Keap1/Nrf2/SOD2 pathway, resulting in the ROS accumulation. This work identified Keap1 alkylation as a new drug target for tuberculosis and provides a preliminary basis for the development of antituberculosis lead compounds based on JFD.

## 1. Introduction

Tuberculosis is a chronic infectious disease caused by *Mycobacterium tuberculosis* infection. In 2019, there were about 10 million new cases of tuberculosis, and tuberculosis caused more than 1.4 million deaths worldwide [1]. Although first-line antibiotic treatments for tuberculosis, including isoniazid, rifampicin, and pyrazinamide, have a 90% success rate, recurrence occurs in up to 9% of cases after treatment completion [2, 3]. More importantly, the number of multidrug-resistant tuberculosis cases is growing rapidly, yet the success rate of antibiotic drugs against multidrug-resistant tuberculosis (MDR-TB) is less than 50% [4, 5]. Therefore, new effective strategies for treating tuberculosis are urgently needed.

All the currently used antituberculosis drugs act directly on *M. tuberculosis*, which not only risks cross-resistance, but also ignores the role of host immunity in combating infection [6]. Host immunity can effectively control *M. tuberculosis* infection [7]. Therefore, host-directed therapy, which aims to improve host immunity against *M. tuberculosis* infection, provides new avenues for tuberculosis treatment [8, 9]. Macrophages are the first line of host defense against *M. tuberculosis* infection [10, 11]. The antituberculosis mechanisms of macrophages mainly include the production of reactive oxygen species (ROS), reactive nitrogen species, and inflammatory factors, as well as autophagy and apoptosis [12, 13]. ROS, which can directly kill *M. tuberculosis* in macrophages without causing host cell death, have a strong oxidizing activity and function as the main effector molecule that eliminates *M. tuberculosis* from macrophages [14, 15]. Indeed, increasing the levels of ROS enhances the elimination of *M. tuberculosis* in macrophages [16, 17]. In macrophages, ROS accumulation is attenuated by the actions of several detoxification and antioxidant enzymes such as superoxide dismutase (SOD), NAD(P)H dehydrogenase (quinone) 1 (NQO1), and glutathione S-transferase (GST) [18–20]. One of the most important regulators of the activity of such enzymes is the nuclear transcription factor erythroid 2-related factor 2 (Nrf2) [21]. Nrf2 acts as a guardian of redox homeostasis and is a promising host therapeutic target for treating oxidative stress-associated diseases [22, 23]. The level and activity of Nrf2 are regulated by the substrate adaptor protein Kelch-like ECH-associated protein 1 (Keap1) [24]. Keap1 cysteine residues are critical sensor for regulating induction of phase 2 enzymes that protect from oxidative stress [25, 26]. During oxidative stress, some key cysteine residues of Keap1 are covalently modified, which finally leads to the dissociation of Nrf2 from Keap1 and accumulation in the nucleus [25–29], where it forms a transcription factor complex and induces gene expression of antioxidant enzymes [30]. Thus, modifying the Keap1-Nrf2 interaction during oxidative stress could be a therapeutic strategy to further inhibit the antioxidant response against oxidative stress. This strategy would lead to excessive accumulation of ROS, thereby enhancing the elimination of *M*. *tuberculosis*.

Traditional medicinal herbs have been used for centuries for preventing and treating diseases [31] and are valuable sources for identifying lead compounds that are then refined into safe and efficacious drugs [32, 33]. As the molecular mechanisms of traditional medicinal herbs are different from antibiotics, they provide new opportunities for the treatment of tuberculosis, especially multidrug-resistant tuberculosis [8]. *Honeysuckle* (the flowers of *Lonicera japonica*) has been used as the local and traditional medicine in clinical practice for the treatment of exopathogenic disease, epidemic febrile diseases, pulmonary tuberculosis, and several infection diseases for almost 1500 years [34]. In previous studies, we investigated the chemical constituents and antipathogen activity of *honeysuckle* [35–37]. We identified the biflavonoid japoflavone D (JFD) from *honeysuckle* and showed that it increases the ROS levels in human hepatocarcinoma cell lines SMMC-7721 and HepG2 by inhibiting the Nrf2 signaling pathway [38]. These findings suggest that JFD may have the potential to kill *M*. *tuberculosis* in host macrophages. Herein, this study uses the human monocyte THP-1 cell line as a model to investigate the effects of JFD on eliminating *M*. *tuberculosis* from macrophages.

## 2. Materials and Methods

### 2.1. Bacterial Strains and Culture Conditions

*M*. *tuberculosis* strains H37Ra, H37Rv, and GFP-labeled H37Ra used in this study were cultured in Middlebrook 7H9 broth (BBL Microbiology Systems) supplemented with 10% oleic acid-albumin-dextrose-catalase (OADC; Becton, Dickinson), 0.05% Tween 80, and 0.2% glycerol. After culturing for 5 to 7 days at 37°C with shaking, the bacteria were resuspended in serum-free RPMI medium and sonicated to obtain a suspension of single cells before use. The concentration of bacteria was determined by a microplate reader.

### 2.2. Cell Lines and Reagents

The human monocytic cell line THP-1 was purchased from the Cell Bank of the Chinese Academy of Sciences (Shanghai, China). THP-1 cells were cultured in RPMI 1640 (Corning, New York, USA) culture medium supplemented with 10% fetal bovine serum (FBS, HyClone, Logan, USA) at 37°C and 5% CO_2_. The THP-1 cells were seeded at 2 × 10^5^ cells/ml in a 24-well plate and differentiated with 40 ng/ml phorbol 12-myristate 13-acetate (PMA, Sigma-Aldrich) for 24 h. Media were then replaced with fresh medium and cells were incubated for 12 h before use. Doramapimod, luteolin, and MitoTEMPO were purchased from MedChemExpress (Shanghai, China). Cycloheximide, chloroquine, NAC, DL-dithiothreitol (DTT), iodoacetamide, formic acid, acetonitrile, methanol, and hoechst were purchased from Sigma-Aldrich (St. Louis, USA). The final concentration of DMSO in treatments did not exceed 0.1% (vol/vol). JFD was purified from *L*. *japonica* flower buds as described in our previous work [35]. Protease, WST1, and FITC annexin V apoptosis detection kits were obtained from Beyotime (Shanghai, China). Recombinant human Keap1 was purchased from Sino Biological (Beijing, China). Anti-human JNK (9252), phoshpho-Thr183/Tyr185 JNK (9255), p38 MAPK (8690), phoshpho-Thr180/Tyr182 p38 MAPK (4511), ERK1/2 (4695), and phoshpho-Thr202/Tyr204-ERK1/2 (4370) antibodies were purchased from Cell Signaling Technology (Danvers, MA, USA). Anti-human cleaved caspase-3 (66470-2-Ig), Nrf2 (16396-1-AP), Keap1 (10503-2-AP), GAPDH (60004-1-lg), *β*-actin (66009-1-Ig), recombinant Nrf2 (Ag9469), and recombinant Keap1 (Ag0779) were purchased from Proteintech (Rosemont, IL, USA).

### 2.3. Cell Viability Assay

Cell viability was measured using the WST-1 assay. Briefly, the cells were seeded at a density of 1.0 × 10^4^ cells/well in a 96-well plate for 24 hours, then treated with DMSO or with different concentrations of JFD for 72 hours. After the treatment, 10 *μ*l of WST-1 was added to each well and the plates were incubated for 2-4 hours. The absorbance (optical density) was measured using a spectrophotometric microtiter plate reader at 450 nM.

### 2.4. CFU Assays and Evaluation of Phagocytosis of H37Ra

PMA-differentiated THP-1 macrophages (2 × 10^5^ cells/well) were infected with H37Ra or H37Rv at a multiplicity of infection (MOI) of 10 for 6 hours at 37°C, 5% CO_2_. Cells were then washed three times with prewarmed PBS to remove extracellular bacteria. Infected THP-1 cells were then treated with drugs as indicated for another 72 hours. The cells were washed three times with PBS then lysed with 500 *μ*l PBS containing 0.1% SDS. Triplicate experimental groups for each treatment were plated on 7H10 agar supplemented with 10% OADC. After incubation at 37°C for 2 weeks, the colonies were counted. To evaluate phagocytosis of H37Ra, GFP-labeled H37Ra was used to infect THP-1 cells at an MOI of 10 for 6 hours. Cells were then washed three times with prewarmed PBS and harvested before quantification by BD FACSAria II flow cytometry and analyzed using FlowJo v10 software.

### 2.5. Apoptosis Assay

Detection of apoptotic cells was performed with a FITC annexin V apoptosis detection kit (C1062L, Beyotime) according to the manufacturer's protocol. Briefly, cells were harvested after drug treatment, washed twice with ice-cold PBS, and incubated with propidium iodide (PI) and annexin V conjugated with FITC for 15 min in the dark. The stained cells were then detected by BD FACSAria II flow cytometry and analyzed using FlowJo v10 software.

### 2.6. Measurement of Caspase-3, -7, and -8 Activity

Caspases-3, -7, and -8 activities were determined by using a Caspase-Glo 3/7 or a Caspase-Glo 8 assay kit (Promega) according to the manufacturer's instructions. In brief, the cells were seeded at a density of 1.0 × 10^4^ cells/well in a 96-well plate. After drug treatment, an equal volume of Caspase-Glo 3/7 or Caspase-Glo 8 reagent was added to the cell culture medium, which had been equilibrated to room temperature for 1 hour. Cells were shaken for 5 min and incubated at room temperature for 30 min. Luminescent recording was performed with a Synergy H1 microplate reader (BioTek).

### 2.7. Measurement of ROS Activity and Mitochondrial Membrane Potential

PMA-differentiated THP-1 macrophages (2 × 10^5^ cells/well) were infected with H37Ra at a MOI of 10 : 1 (in the presence or absence of JFD) for 6 or 24 hours. Extracellular bacteria were removed by washing three times with prewarmed PBS. The generation of cytoplasmic ROS (cROS), mitochondrial ROS (mROS), and the mitochondrial membrane potential was assessed by staining with CM-H2DCF-DA (Invitrogen, Carlsbad, USA), MitoSOX red mitochondrial superoxide indicator (Invitrogen, Carlsbad, USA), and MitoTracker red (Invitrogen, Carlsbad, USA), respectively, according to the manufacturer's instructions. The fluorescence intensities of the cells were measured by BD FACSAria II flow cytometry and analyzed by FlowJo v10 software.

### 2.8. Quantitative PCR (qPCR)

qPCR was performed as previously described [39]. The mRNA levels of target genes were normalized to GAPDH levels and further normalized to the negative control. The fold changes in gene expression were calculated through relative quantification (2^−ΔΔCt^). The primer sequences used to amplify the target genes are shown in Supplementary Material Table S1.

### 2.9. RNA Interference and Transfection

PMA-differentiated THP-1 macrophages (2 × 10^5^ cells/well) were transfected with SOD2 siRNA (RiboBio, Guangzhou, China) using Lipofectamine RNAiMAX (Thermo Fisher, Waltham, USA) according to the manufacturer's protocol. After transfection for 48 hours, the cells were washed once with PBS before lysing and the efficiency of gene silencing was determined by real-time quantitative PCR and western blotting. To count CFUs, THP-1 cells were infected with H37Ra (MOI of 10 : 1) 48 hours after transfection. siRNA-silenced cells were then used in experimental procedures as described above.

### 2.10. Analysis of SOD2 Transcription Levels in Clinical Samples

The participants were recruited from the Third People's Hospital of Shenzhen (Shenzhen, China) in 2019, including 46 healthy control and 62 tuberculosis patients. This experimental scheme was approved by the Ethical Review Committee of Shenzhen People's Hospital (20190301-15). Human peripheral blood mononuclear cells (PBMCs) isolated from whole blood were then sent to BGI Genomics Co., Ltd. (Shenzhen, China) to do transcriptome sequencing. The expression levels of SOD2 in different samples were analyzed.

### 2.11. Immunofluorescent Staining

Cells cultured on coverslips were washed with PBS and fixed with 4% paraformaldehyde for 10 min after being treated as indicated. The cells were then incubated with PBS containing 0.25% Triton X-100 to permeabilize the membrane, then blocked with 5% BSA for 1 hour. Thereafter, the cells were incubated with specific primary antibodies overnight at 4°C. The coverslips were then washed three times with PBS, followed by incubation for 1 hour in the dark with fluorescent secondary antibodies. Coverslips were stained with Hoechst for 10 min and immunofluorescence was measured by a fluorescence microscope.

### 2.12. Western Blot Analysis

To obtain total protein, cells were lysed in SDS loading buffer (20 mM Tris, 150 mM NaCl, 1% Triton X-100, 1 mM EDTA, 1 mM EGTA, and 0.1% SDS) supplemented with 1 mM PMSF. To extract nuclear and cytoplasmic protein, cells were lysed using a nuclear and cytoplasmic protein extraction kit (Beyotime) according to the manufacturer's instructions. The concentration of protein was measured using BCA reagent (Beyotime). SDS-PAGE and western blotting were performed as previously described [25].

### 2.13. ChIP Assay

Chromatin immunoprecipitation (ChIP) assays were performed following the protocol of Upstate Biotechnology's ChIP assay kit (17-10085). In brief, interrupted chromatin was immunoprecipitated using an anti-Nrf2 antibody (16396-1-AP, Proteintech) as recommended by the manufacturer. Rabbit IgG was used as a negative control. DNA purified from immunoprecipitated and preimmunoprecipitated samples was diluted 1 : 100 and subjected to PCR amplification using the following primers for the SOD2 promoter region: forward 5′-tgctccccgcgctttcttaag-3′; reverse 5′-gctgccgaagccaccacag-3′.

### 2.14. Molecular Docking

A molecular docking study was performed to investigate the interaction between JFD and Keap1 using the AutoDock Vina 1.1.2 package [40]. The three-dimensional (3D) coordinate of Keap1 (PDB ID: 5WFV) was retrieved from the RCSB Protein Data Bank. The 2D structure of JFD was drawn using ChemBioDraw Ultra 14.0 and converted to the 3D structure and optimized using the MMFF94 force field algorithm. The AutoDockTools 1.5.6 package was employed to generate the docking input files [41, 42].

### 2.15. Fluorescence Spectroscopy

Human recombinant Keap1 protein was dissolved in 50 mM PBS (pH 7.6) at a concentration of 0.1 mg/ml. Successively increasing concentrations of JFD were added to the Keap1 protein solution, and a BioTek Synergy H1 fluorescence microplate reader was to detect changes in the fluorescence spectra and the light absorbed by the Keap1 protein solution after excitation at 280 nM. The fluorescence quenching rate constant *K*_*q*_ was calculated using the formula: *F*_0_/*F* = 1 + *K*_*q*_*τ*_0_[*Q*].

### 2.16. Immunoprecipitation

PMA-differentiated THP-1 macrophages (2 × 10^7^ cells) were infected with H37Ra at an MOI 10 : 1 (in the presence or absence of JFD) for 24 hours then lysed with RIPA lysis buffer. The Keap1 antibody and 4 *μ*g of immunomagnetic beads were added to the lysis buffer. Rabbit IgG was used as a negative control. After overnight incubation, the beads were washed in PBS three times, eluted with SDS loading buffer, and detected using western blotting as described above.

### 2.17. Identification of Keap1 Alkylation Sites with LC-MS/MS Analysis

The identification of Keap1 alkylation sites was performed as described before [25]. Briefly, recombinant human Keap1 (10 *μ*g) was incubated with 1 *μ*M of CDDO-EA in 100 *μ*l of recombinant protein storage buffer (50 mM Tris-HCl, 10 mM reduced glutathione, and pH 8.0) at the presence of JFD or not for 2 h at room temperature. The reaction was quenched by adding 60 *μ*M DTT, and incubated for another 15 min. The mixture was then purified by gel electrophoresis using established protocol, followed by in gel-digestion. The tryptic peptides were analyzed by a Q Exactive Hybrid Quadrupole-Orbitrap mass spectrometer (Thermo Fisher Scientific, Waltham, USA). The raw MS file was analyzed and searched against target protein database based on the species of the sample using Byonic.

### 2.18. Pharmacokinetic Study

The pharmacokinetic study protocols were approved by the Experimental Animal Ethics Review Committee of Jinan University (20210301-15, Guangzhou, China). A C57BL/6J mouse, 8 weeks old and weighing 20 g, was put into a metabolic cage and fasted for 12 h, but free to water before dosing. The mouse was injected via tail vein with JFD at a dose of 20 mg/kg. Approximately, 50 *μ*l blood samples were collected via shearing tail at 0.167, 1, 2, 4, 8, 12, 24, and 48 h after intravenous administration. Plasma was centrifugated at 3000 rpm for 10 min. The urine and feces samples were collected for 12 h before dosing and for 48 h after dosing, respectively. All the above samples are analyzed by HPLC.

### 2.19. Statistical Analysis

Data were analyzed using GraphPad Prism 6.0 software (GraphPad Software Inc., San Diego, CA, USA). The differences between the two groups were estimated with the 2-tailed unpaired *t* test. Quantitative data are reported as means ± SD taken from at least three independent experiments. *p* values of less than 0.05 were considered statistically significant.

## 3. Results

### 3.1. JFD Promotes *M*. *Tuberculosis* Clearance in THP-1 Cells

**Figure 1:**
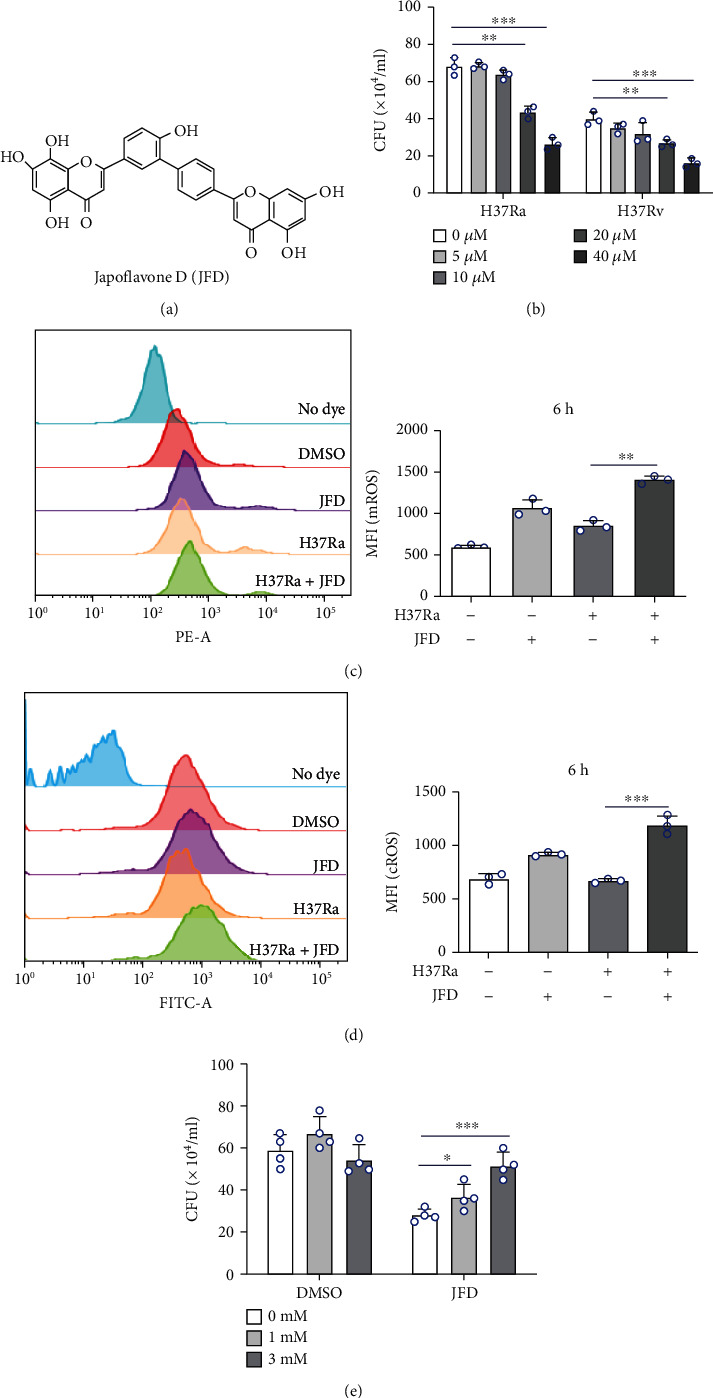
JFD promotes *M. tuberculosis* clearance in THP-1 cells by upregulating ROS. (a) The chemical structure of JFD. (b) The effects of different concentrations of JFD on the survival of *M. tuberculosis* in THP-1 cells. (c, d) The effects of JFD treatment for 6 hours on the levels of mROS and cROS in H37Ra-infected THP-1 cells. (e) The effect of NAC treatment for 24 hours on the antituberculosis activity of JFD. JFD: japoflavone D; *M. tuberculosis*: *Mycobacterium tuberculosis*; CFU: clone-forming units; MFI: mean fluorescence intensity; NAC: N-acetyl-L-cysteine. ^∗^*p* < 0.05, ^∗∗^*p* < 0.01, and ^∗∗∗^*p* < 0.001 compared with the control group. Graphs show the mean ± SD of triplicate wells and are representative of three independent experiments.

Our previous work showed that JFD increases ROS levels in human SMMC-7721 and HepG2 cells [38]. Since ROS is involved in *Mycobacterium tuberculosis* infection control in macrophages, we investigated the effect of JFD on *M*. *tuberculosis* clearance in THP-1 cells. H37Ra, a virulence-attenuated strain of *M*. *tuberculosis* [43], was used as a model strain in this study. JFD (Figure 1(a)) significantly decreased the amount of H37Ra in THP-1 cells in a concentration-dependent manner (Figure 1(b)) without significantly affecting cell viability (Figure S1A). To verify the antibacterial activity of JFD against different *M*. *tuberculosis* strains, we used H37Rv, a virulent type strain of *M*. *tuberculosis* [43], to infect THP-1 cells. A similar effect was observed in THP-1 cells infected with H37Rv (Figure 1(b)). JFD did not affect the growth of H37Ra in 7H9 medium (Figure S1B), nor the phagocytosis of H37Ra (Figure S1C). Taken together, these results indicate that JFD promotes *M*. *tuberculosis* clearance in THP-1 cells, but the molecular mechanism of antituberculosis activity of JFD is still unclear.

ROS are the main effector molecules that eliminate *M*. *tuberculosis* from macrophages [15, 44]. We investigated the effect of JFD treatment on ROS levels in H37Ra-infected THP-1 cells. As shown in Figures 1(c)–1(d), JFD increased both cellular and mitochondrial ROS accumulation after 6 hours treatment in H37Ra-infected THP-1 cells. These data suggest that JFD induced ROS accumulation, which may be responsible for the antituberculosis activity of JFD. To confirm this hypothesis, we used the ROS scavenger NAC to prevent JFD-induced ROS accumulation. As expected, NAC concentration dependently reversed the antituberculosis effect of JFD (Figure 1(e)). Autophagy and the production of inflammatory factors are additional antituberculosis mechanisms, so we investigated the effects of JFD treatment on autophagy and secretion of inflammatory factors (TNF*α* and IL-1*β*). However, the results showed that JFD did not affect the secretions of TNF*α* and IL-1*β* (Figure S2A-B) nor autophagy of H37Ra-infected THP-1 cells (Figure S2C). Furthermore, the autophagy inhibitors chloroquine and 3-methyladenine had no effects on the antituberculosis activity of JFD (Figure S2D). Together, JFD promotes *M*. *tuberculosis* clearance in THP-1 cells through upregulating levels of ROS.

### 3.2. JFD Induces Apoptosis through ROS-Mediated p38 Signaling Activation in H37Ra-Infected THP-1 Cells

**Figure 2:**
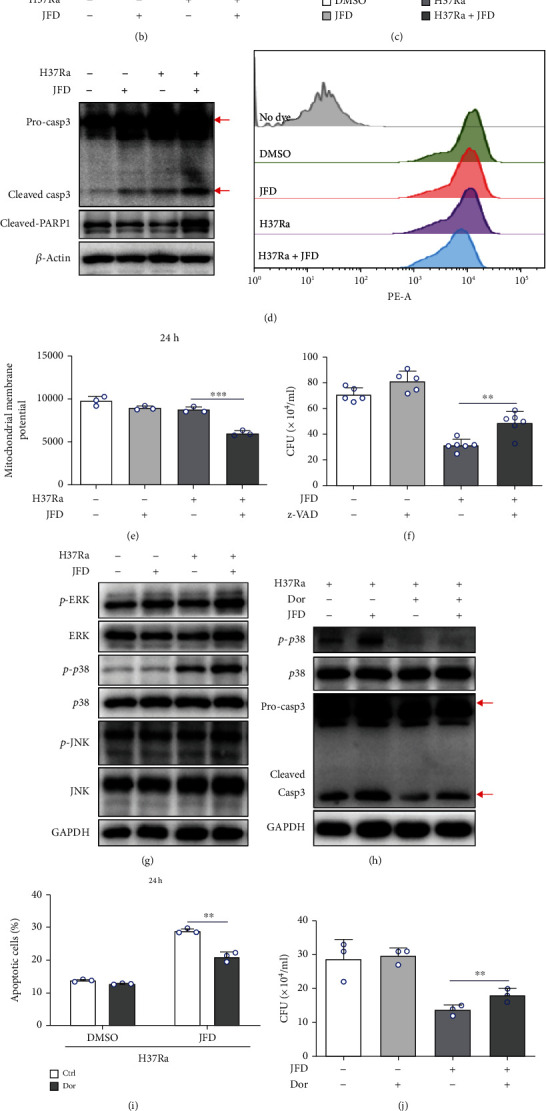
JFD induces apoptosis in H37Ra-infected THP-1 cells through ROS-mediated p38 signaling activation. The effects of JFD treatment for 24 hours on (a, b) apoptosis, (c) caspase-3, -7, and -8 activity, (d) caspase-3 activation, and (e) the mitochondrial membrane potential of H37Ra*-*infected THP-1 cells. (f) The effect of z-VAD treatment for 24 hours on the antituberculosis activity of JFD. (g) Western blot analysis to detect the effect of JFD treatment for 24 hours on the activation of the MAPK signaling pathway in H37Ra-infected THP-1 cells. The effect of doramapimod treatment for 24 hours on (h) caspase-3 cleavage, (i) apoptosis in H37Ra-infected THP-1 cells, and (j) the antituberculosis activity of JFD in H37Ra-infected THP-1 cells. The effect of NAC treatment for 24 hours on (k) caspase-3 cleavage and (l) apoptosis in H37Ra-infected THP-1 cells. JFD: japoflavone D; CFU: clone-forming units; NAC: N-acetyl-L-cysteine; z-VAD: z-VAD-fmk; Dor: doramapimod. ^∗^*p* < 0.05, ^∗∗^*p* < 0.01 compared with the control group, and ^∗∗∗^*p* < 0.001 compared with the control group. Graphs show the mean ± SD of triplicate wells and are representative of three independent experiments.

Cell death induced by elevated ROS levels may contribute to the antituberculosis effect of JFD [45], so we determined the effect of JFD treatment on the survival of H37Ra-infected THP-1 cells. The results showed that JFD treatment for 24 hours increased apoptotic cell death (Figures 2(a) and 2(b)) and induced the activities of proapoptotic caspase-3, -7, and -8 in H37Ra-infected THP-1 cells (Figure 2(c)). The induction of cleaved caspase-3 and PARP1 was confirmed by western blotting (Figure 2(d)). Flow cytometry analysis showed that JFD treatment shifted the mitochondrial membrane potential to a more negative value (Figure 2(e)). These data show that JFD treatment increases apoptosis of H37Ra-infected THP-1 cells. To test whether the induction of apoptosis contributes to the antituberculosis activity of JFD, we used the caspase inhibitor z-VAD-fmk [46] to inhibit JFD-induced apoptosis and then quantified the amount of H37Ra in THP-1 cells post H37Ra infection and JFD treatment for 72 hours. As shown in Figure 2(f), z-VAD-fmk reduced the antituberculosis activity of JFD, which suggests that the induction of apoptosis partially contributes to the antituberculosis activity of JFD.

Apoptosis is regulated by a complex signaling network, which includes the AKT-mTOR and MAPK signaling cascades [47, 48]. So, we detected the activation of these two pathways. As shown in Figure 2(g) and Figure S3, JFD treatment specifically activated p38 MAPK signaling, but did not alter the activation of AKT-mTOR, JNK, or ERK proteins. To validate whether p38 MAPK activation is required for JFD-induced apoptosis in H37Ra-infected THP-1 cells, we assessed the cleavage of caspase-3 after JFD treatment in the presence of doramapimod, a potent p38 MAPK inhibitor [49]. As expected, doramapimod abolished JFD-induced caspase 3 cleavage (Figure 2(h)). Consistently, flow cytometry analysis confirmed that doramapimod treatment suppressed JFD-induced apoptosis in H37Ra-infected THP-1 cells (Figure 2(i)). Moreover, doramapimod treatment partially reversed the JFD-induced clearance of H37Ra from infected THP-1 cells (Figure 2(j)). Many studies indicate that ROS can activate p38 MAPK signaling [50, 51], so we determined the effect of NAC on JFD-induced p38 activation and apoptosis in H37Ra infected THP-1 cells. Not surprisingly, the NAC treatment reversed ROS-induced p38 activation and apoptosis, which is consistent with published reports (Figures 2(k) and 2(l)). In summary, JFD induces apoptosis by activating ROS-mediated p38 signaling in H37Ra-infected THP-1 cells.

### 3.3. JFD Promotes ROS Accumulation by Inhibiting SOD2 Transcription

**Figure 3:**
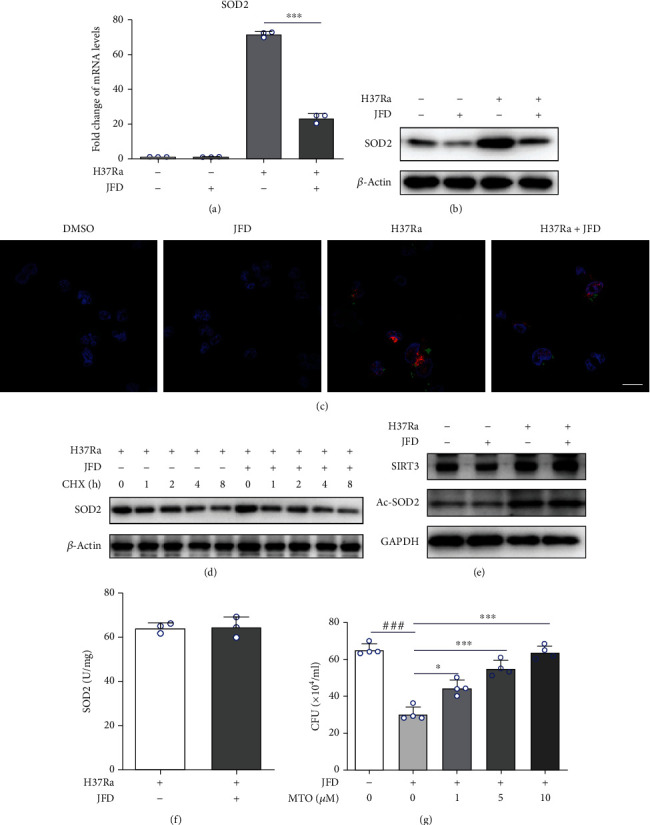
JFD promotes ROS accumulation by inhibition of SOD2 transcription. (a) qPCR, (b) western blot, and (c) immunofluorescence analysis of the effect of JFD treatment for 24 hours on SOD2 expression levels in H37Ra-infected THP-1 cells. The effect of JFD treatment on the (d) stability, (e) acetylation, and (f) enzymatic activity of the SOD2 protein. (g) The effect of MitoTEMPO treatment for 24 hours on the antituberculosis activity of JFD. JFD: japoflavone D; CFU: clone-forming units; CHX: cycloheximide; MTO: MitoTEMPO. The scale bar represents 5 *μ*m. ^∗^*p* < 0.01, ^∗∗^*p* < 0.01, and ^∗∗∗^*p* < 0.001 compared with the control group. Graphs show the mean ± SD of triplicate wells and are representative of three independent experiments.

Superoxide dismutase (SOD) catalyzes the conversion of superoxide anions into hydrogen peroxide and is an important regulator of oxidative stress [52, 53]. We investigated the effect of JFD treatment on the expression levels of SOD1 (found predominately in intracellular cytoplasmic spaces), SOD2 (found predominately in mitochondria), and SOD3 (found exclusively in extracellular spaces). JFD treatment suppressed H37Ra infection-induced increases in SOD2 expression (Figures 3(a) and 3(b)); this effect was confirmed by immunofluorescence experiments (Figure 3(c)). JFD did not significantly alter the expression of SOD1 or SOD3 (Figure S4). To determine the effect of JFD treatment on the stability of SOD2, we used cycloheximide to block protein synthesis. As shown in Figure 3(d), JFD treatment did not affect the stability of SOD2. In addition, JFD treatment did not affect the acetylation (Figure 3(e)) or enzymic activity (Figure 3(f)) of SOD2. We next used MitoTEMPO, a scavenger of mitochondrial ROS, to confirm the role of SOD2 in ROS-mediated clearance of H37Ra induced by JFD treatment [54]. In agreement with the results described above, MTO reversed the antituberculosis effect of JFD (Figure 3(g)). Taken together, these results show that inhibition of H37Ra-induced SOD2 transcription is a vital mechanism underlying the antituberculosis effect of JFD.

### 3.4. H37Ra Infection Induces SOD2 Expression to Eliminate Excess ROS

**Figure 4:**
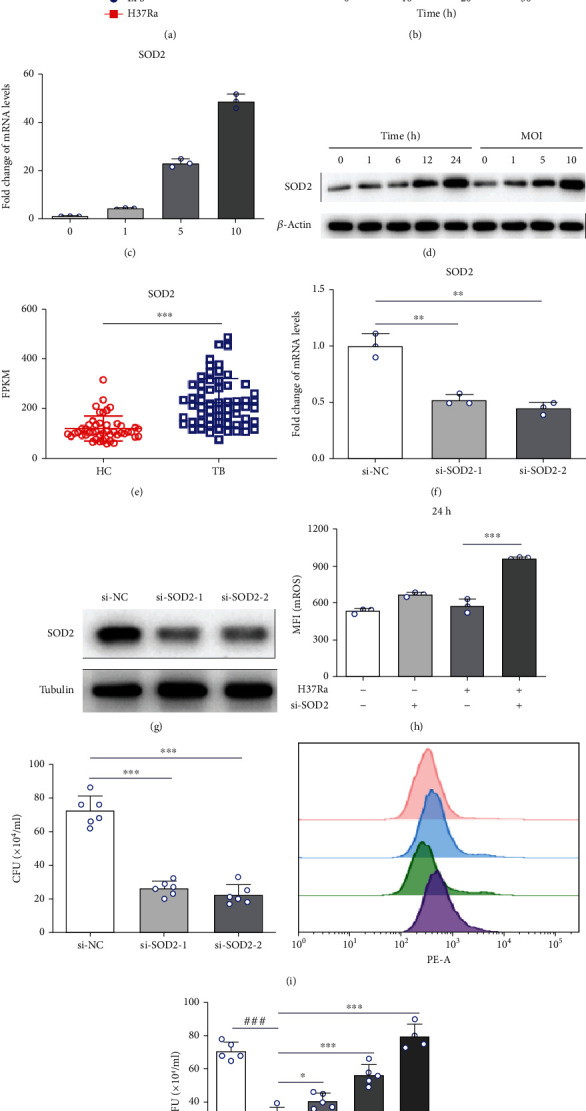
H37Ra infection induces SOD2 expression to eliminate redundant ROS. (a) Transcriptome sequencing to detect changes in SOD2 transcription levels after LPS or H37Ra stimulation in THP-1 cells. qPCR detection of changes in SOD2 transcription levels associated with (b) H37Ra infection time or (c) multiplicity of infection in THP-1 cells. (d) Western blot analysis of changes in SOD2 expression levels associated with H37Ra infection time or multiplicity of infection in THP-1 cells. (e) Transcriptome sequencing to detect SOD2 transcription levels in peripheral blood mononuclear cells from healthy controls and TB patients. (f) qPCR and (g) western blot analysis to confirm RNA silencing of SOD2. The effect of SOD2 silencing on mROS production (h) and H37Ra survival (i) in THP-1 cells. (j) The effects of different concentrations of MTO on the antituberculosis effect of SOD2 silencing. JFD: japoflavone D; MTO: MitoTEMPO; MOI: multiplicity of infection; CFU: clone-forming units; MFI: mean fluorescence intensity; TB: tuberculosis; HC: healthy control; FPKM: fragments per kilobase per million. ^∗^*p* < 0.05, ^∗∗^*p* < 0.01, and ^∗∗∗^*p* < 0.001 compared with the control group. Graphs show the mean ± SD of triplicate wells and are representative of three independent experiments.

To confirm the role of SOD2 in *M*. *tuberculosis* survival, we used our previously generated mRNA sequencing data to determine the expression level of SOD2 in macrophages stimulated with LPS or H37Ra infection. As shown in Figure 4(a), H37Ra infection, but not LPS, significantly increased SOD2 expression. This result was confirmed by qPCR and western blot analysis of H37Ra-infected THP-1 cells (Figures 4(b)–4(d)). In addition, we compared the expression level of SOD2 in PBMCs from TB patients and healthy controls using our previous mRNA sequencing data. SOD2 expression was higher in TB patients than in healthy controls (Figure 4(e)), indicating that SOD2 expression was induced specifically by H37Ra infection. We next constructed SOD2-silenced THP-1 cells with siRNA-mediated knockdown (Figures 4(f) and 4(g)). As shown in Figures 4(h) and 4(i), impaired SOD2 expression caused mROS accumulation and promoted H37Ra clearance from THP-1 cells. Furthermore, MitoTEMPO treatment reversed the antituberculosis effect of SOD2 silencing (Figure 4(j)). In summary, H37Ra infection greatly increases SOD2 expression to eliminate excess ROS and promote the survival of H37Ra in THP-1 cells. This indicated that SOD2 was crucial for *M*. *tuberculosis* to escape the killing of macrophages, which make SOD2 a potential target of antituberculosis drugs.

### 3.5. JFD Suppresses SOD2 Transcription by Interrupting Nuclear Transport of Nrf2

**Figure 5:**
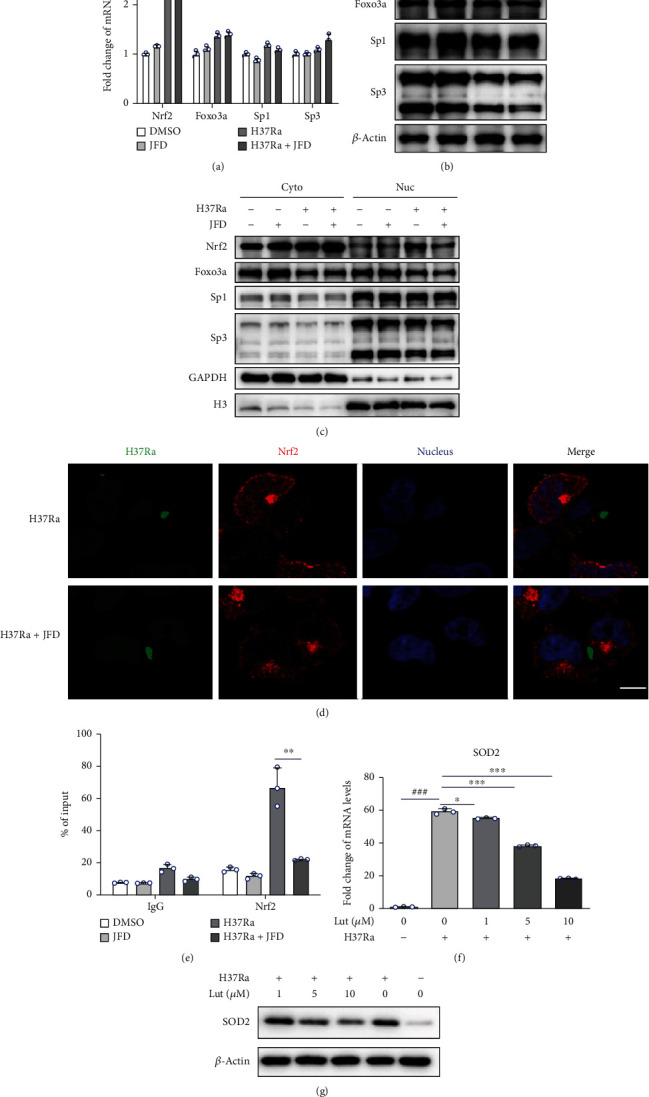
JFD inhibits the nuclear localization of Nrf2. (a) qPCR and (b) western blot analysis were used to detect the effect of JFD treatment for 24 hours on the expression of transcription factors that regulate SOD2 activity. (c) Western blot and (d) immunofluorescence experiments were used to detect the effect of JFD treatment for 24 hours on the translocation of transcription factors. (e) ChIP analysis was used to determine the effect of JFD treatment for 24 hours on the binding of Nrf2 to the SOD2 promoter region. (f) qPCR and (g) western blot analysis were used to detect the effect of different concentrations of Lut treatment for 24 hours on SOD2 expression. JFD: japoflavone D; Cyto: cytoplasmic; Nuc: nuclear; Lut: luteolin. ^∗^*p* < 0.01, ^∗∗^*p* < 0.01, and ^∗∗∗^*p* < 0.001 compared with the control group. Graphs show the mean ± SD of triplicate wells and are representative of three independent experiments.

To uncover the mechanism underlying the inhibitory effect of JFD on SOD2 transcription, we analyzed the expression level of the transcription factors Nrf2, Foxo3a, Sp1, and Sp3, which are responsible for SOD2 transcription [55]. Surprisingly, JFD treatment did not affect the expression of these transcription factors (Figures 5(a) and 5(b)). Therefore, we determined if JFD treatment altered the nuclear localization of these transcription factors in H37Ra-infected THP-1 cells after nuclear and cytoplasmic separation. As shown in Figure 5(c), JFD inhibited H37Ra infection-induced Nrf2 nuclear localization, but did not affect the localization of Foxo3a, Sp1, or Sp3. These results were confirmed by immunofluorescence experiments (Figure 5(d)). Additionally, ChIP analysis demonstrated that JFD treatment interrupted the binding of Nrf2 to the transcription start site of SOD2 (Figure 5(e)). Importantly, the Nrf2 inhibitor luteolin [56] reduced H37Ra infection-induced increases in SOD2 expression (Figures 5(f) and 5(g)). In summary, these observations indicate that JFD treatment suppressed H37Ra infection-induced SOD2 transcription by interrupting the nuclear transport of Nrf2.

### 3.6. JFD Targets Keap1 to Distrube the Alkylation of Cysteines on Keap1

**Figure 6:**
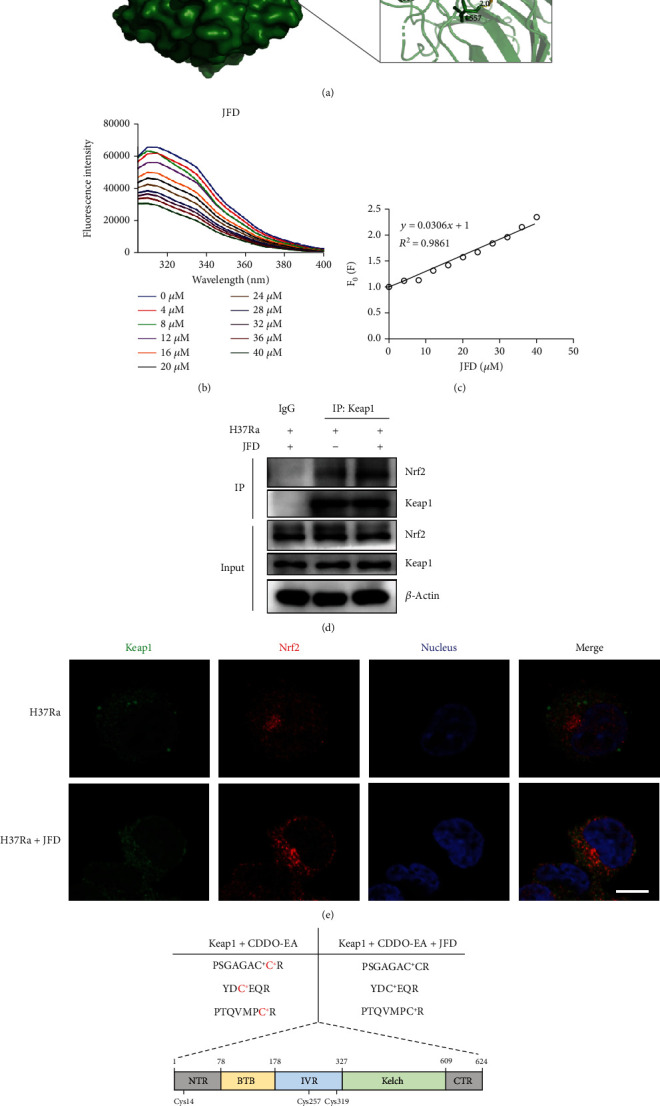
JFD targets Keap1 to inhibit the alkylation of Keap1. (a) A simulated interaction binding mode between JFD and the Keap1-Nrf2 protein complex. The effect of JFD on (b) the autofluorescence of the recombinant Keap1 protein and (c) the fluorescence quenching rate constant *K*_*q*_. (d) Immunoprecipitation and (e) immunofluorescence experiments were used to detect the effect of JFD treatment for 24 hours on the interaction between Keap1 and Nrf2. (f) The effect of JFD on CDDO-EA-induced alkylation of cysteines on Keap1. JFD: japoflavone D; C^+^: carbamidomethylated cysteine; C^∗^: CDDO-EA-modified cysteine.

The Keap1/Nrf2 pathway is one of the most crucial antioxidant protection mechanisms in cells [57]. During oxidative stress, the transcription factor Nrf2 dissociates from Kelch-like ECH-related protein 1 (Keap1), which causes the Nrf2 move from the cytosol to the nucleus to promote the transcription of downstream genes [58]. To explore the mechanism by which JFD interrupts the nuclear transport of Nrf2, we imitated the interaction between JFD and Nrf2 or Keap1 using the molecular docking analyzing software AutoDock. The results showed that JFD has the potential to bind to Keap1 with ΔGb value of -12.1 kcal/mol and that JFD targets Keap1 at Leu365, Ser508, Gln530, and Leu557 residues (Figure 6(a)). To confirm a direct interaction between JFD and Keap1, we detected changes in the fluorescence spectra of the Keap1 protein in solution after excitation at 280 nM when JFD was added successively to the Keap1 protein solution. We observed that the fluorescence gradually quenched with the continuous addition of JFD (Figure 6(b)). The fluorescence quenching rate constant *K*_*q*_ value was 3.06 × 10^12^ (Figure 6(c)), which showed that JFD may directly bind to Keap1. To verify the effect of JFD treatment on the interaction between Keap1 and Nrf2, we employed immunoprecipitation to quantify the amount of Nrf2 that bound to Keap1 after treatment with JFD for 24 hours. As shown in Figure 6(d), JFD treatment stabilized the interaction between Keap1 and Nrf2, which was confirmed by immunofluorescence experiments (Figure 6(e)). Furthermore, LC-MS/MS analysis demonstrated that JFD inhibited CDDO-EA, an representative Michael acceptor, induced alkylation of the Cys14, Cys257, and Cys319 residues on Keap1 (Figure 6(f)), which were critical for the Nrf2 activation [28]. Representative MS/MS secondary spectrums of the peptide containing the CDDO-EA-modified Cys14, Cys257, and Cys319 were shown in Figure S5. In conclusion, JFD targets Keap1 and inhibits the covalent modification of the cysteine residues, which hindered the dissociation of Nrf2 from Keap1 protein complex, thereby interrupting the nuclear transport of Nrf2.

### 3.7. JFD Can Stay as the Prototype for a Long Time in the Mice

In order to preliminarily analyze the druggability of JFD, we studied the pharmacokinetic characteristics of JFD in mice. As shown in Figure S6, JFD can still be detected 24 hours after injection, which indicates that JFD can exist in mice stably for a long time. Moreover, JFD is still mainly in the form of prototypes in mice, which can provide a certain degree of possibility for JFD to exert its efficacy *in vivo*. In addition, JFD was also detected in feces samples after administration (Figure S6), which suggests that JFD can be excreted in feces, and the possibility of accumulation in the body causing toxicity is low.

## 4. Discussion

**Figure 7:**
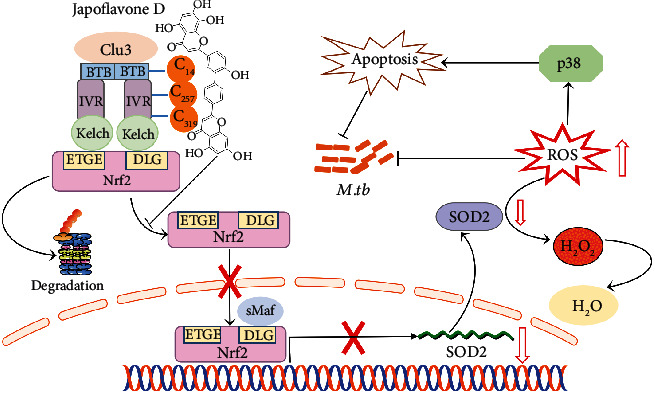
Schematic diagram of JFD's antituberculosis mechanism.

In this study, we showed that JFD enhances the elimination of *M. tuberculosis* by boosting the levels of ROS in macrophages. The massive increase in ROS levels activates p38 signaling to induce apoptosis. JFD suppresses the nuclear transport of Nrf2, thereby inhibiting the transcription of downstream SOD2, leading to a large accumulation of ROS and enhancing the clearance of *M. tuberculosis* in macrophages. Furthermore, we proved that JFD directly targets Keap1 and disturbe the alkylation of several key cysteine residues Cys14, Cys257, and Cys319, which were reported to be crucial for the nuclear transport of Nrf2. In pharmacokinetic study, JFD can stay as the prototype for 24 h in mice and can be excreted in feces without any toxicity. The involved mechanisms are illustrated in Figure 7.

ROS have strong oxidizing effects, which can directly kill *M. tuberculosis* in macrophages without causing host cell death [59]. Indeed, ROS are the main effector molecules that eliminate *M. tuberculosis* from macrophages. Ouyang et al. reported that the estrogen receptor modulator bazedoxifene enhances the elimination of *M. tuberculosis* in macrophages by promoting ROS production [16], showing that it is possible for a drug to increase the levels of ROS and mediate *M. tuberculosis* clearance. Our results showed that JFD enhanced the elimination of *M. tuberculosis* and increased both cellular and mitochondrial ROS accumulation in H37Ra-infected THP-1 cells. Moreover, the ROS scavenger NAC reversed the antituberculosis effect of JFD, which suggest that JFD-induced ROS accumulation may directly contribute to its antituberculosis activity. In the meantime, large increases in ROS levels induced by JFD can activate p38 signaling to enhance apoptosis, thereby enhancing H37Ra clearance.

The production of ROS in the cell is in a delicate balance with the antioxidant defense mechanism. In mammalian cells, three distinct isoforms of SOD enzymes control the formation of ROS. Here, we reported that JFD suppresses H37Ra-induced SOD2 transcription, and SOD2 deficiency enhances host responses to *M. tuberculosis* infection by blocking ROS elimination in macrophages. Although the role of SODA and SODC proteins in *M. tuberculosis* survival in macrophages has been widely studied [60–62], ours is the first report of the involvement of SOD2. SOD2 activity was regulated at the transcriptional, posttranscriptional, and posttranslational level, which makes SOD2 a promising new target for tuberculosis treatment.

Several studies show that Nrf2 regulates the transcription of SOD2 [63, 64]. More importantly, it has been reported that Nrf2 plays an important role in TB infection [65]. For example, tuberculosis-infected Nrf2-deficient mice have a significantly reduced *M. tuberculosis* load and granuloma formation [66, 67], but the specific mechanism underlying this effect is unclear. Furthermore, sulforaphane, a well-established activator of Nrf2, was reported to trigger apoptosis and inhibited the mycobacterial growth [68]. Given the important role of Nrf2 signaling in ROS regulation, Nrf2 could be a therapeutic target for the development of antituberculosis drugs. Although reports about drug research or lead compounds targeting Nrf2 for the treatment of tuberculosis do not exist, many Nrf2 inhibitors have been reported [69]. These inhibitors include (1) nuclear receptor agonists, such as dexamethasone, clobetasol propionate, and bexarotene; (2) natural compounds, such as luteolin, wogonin, brusatol, mycotoxin aspergillus toxin A, the coffee alkaloid trigonelline, and ascorbic acid; (3) small-molecule compounds such as ML385. The targets, mechanism of action, and potential therapeutic effects of such inhibitors on tuberculosis are unclear, and the molecules are likely to be associated with poor specificity and considerable side effects. Because treatment of tuberculosis requires long-term medication, molecules that targeted Nrf2 would need to be safe and nontoxic. *Honeysuckle*, from which JFD was isolated, is one of the first varieties listed as both edible and medicinal in resources authorized by the Ministry of Health of China [35]. In our previous study, we showed that JFD inhibits the Nrf2 signaling pathway without major side effects [38]. And JFD treatment suppressed H37Ra infection-induced SOD2 transcription through interrupting nuclear transport of Nrf2. Recently, Petrillo et al. reported the personalized profiles of antioxidant signaling pathway in patients with tuberculosis, and TB treatment strategies based on changing the redox environment in vivo should be individually designed according to the characteristics of each patient and the degree of disease progression as their variability of Nrf2, GSH, HO1, and SOD expression levels [70].

Nrf2 was negatively regulated by its repressor protein Keap1, which binds to Nrf2 in the cytosol and targets it for ubiquitination and proteasomal degradation. Keap1 also acts as the sensor and switch for the Nrf2 ubiquitination machine [71]. Our molecular docking results showed that JFD may bind to Keap1 at Leu365, Ser508, Gln530, and Leu557 residues, and we demonstrated the direct interaction between JFD and Keap1 by fluorescence spectroscopy. Human Keap1 is a cysteine-rich protein that contains 27 cysteine residues, some of which (such as Cys151, Cys257, Cys273, Cys288, Cys297, Cys434, and Cys613) act as sensors of electrophilic and/or oxidative assault [72, 73]. Under stress conditions, these sensitive cysteine residues are oxidized to disulfides or conjugated to electrophiles [74]. These covalent modifications affect the precise assembly of the E3 ligase complex and suppress the ubiquitination of Nrf2, which finally leads to the activation of Nrf2 [75, 76]. To check the effect of JFD on the covalent modifications of cysteine residues on Keap1, we identified the alkylated cysteine residues modified by the classical Michael receptor CDDO-EA through LC-MS/MS. As expected, JFD suppressed the alkylation of Cys14, Cys257, and Cys319 on Keap1, which were reported to be crucial for Nrf2 to dissociate from Keap1-Clu3 complex [77]. Thereby, JFD stabilized the interaction between Nrf2 and Keap1 and interrupting the nuclear transport of Nrf2. Moreover, the effect of JFD on the interaction between Nrf2 and Keap1 was confirmed by immunoprecipitation and immunofluorescent staining. Activation of the Nrf2-regulated cytoprotective system promotes cytosolic reductase activities, elevates the reduced glutathione level, and accelerates the metabolism and export of xenobiotics [78]. The activity of Nrf2 can be regulated by modulating the interaction between Nrf2 and Keap1. The currently known ARE activators are basically indirect inhibitors of Keap1-Nrf2 interaction, and they can form covalent adducts with the sulfhydryl group on Keap1 cysteine through oxidation or alkylation [79]. However, there are no inhibitors of Keap1 alkylation to be reported so far.

## 5. Conclusion

In conclusion, our data reveal for the first time that a novel biflavonoid JFD can suppress the nuclear transport of Nrf2 by acting as a potent inhibitor of Keap1 alkylation. And this is the first research to report the inhibitor of Keap1 alkylation. Furthermore, JFD robustly promotes *M. tuberculosis* elimination from macrophages through inhibiting Keap1/Nrf2/SOD2 signaling axis, which result in the accumulation of ROS. In pharmacokinetic study, JFD can stay as the prototype for 24 h in mice and can be excreted in feces without any toxicity. This work identifies Keap1 alkylation as potential new drug target for tuberculosis and provides a preliminary basis for the development of antituberculosis lead compounds based on JFD.

## Data Availability

The data used to support the findings of this study are available from the corresponding author upon request.

## Abbreviations

ARE: Antioxidant response element
AKT: Serine/threonine kinase 1
CHX: Cycloheximide
cROS: Cellular ROS
ChIP: Chromatin immunoprecipitation
CFU: Clone-forming units
CHQ: Chloroquine
Dor: Doramapimod
DAPI: 4-6-diamidino-2-phenylindole
ERK: Extracellular-regulated protein kinases
Foxo3a: Forkhead box O3A
JFD: Japoflavone D
FBS: Fetal bovine serum
FPKM: Fragments per kilobase per million
HC: Healthy control
HDT: Host-directed therapy
JNK: C-Jun N-terminal protein kinase
Keap1: Kelch-like ECH-associated protein 1
Lut: Luteolin
MDR-TB: Multidrug-resistant tuberculosis
MOI: Multiplicity of infection
MFI: Mean fluorescence intensity
3-MA: 3methyladenine
MTO: MitoTEMPO
OD: Optical density
mTOR: Mammalian target of rapamycin
mROS: Mitochondrial ROS
NAC: N-acetyl-L-cysteine
Nrf2: Nuclear factor erythroid 2-related factor 2
OADC: Oleic acid-albumin-dextrose-catalase
PI: Propidium iodide
PMA: Phorbol 12-myristate 13-acetate
PBMC: Peripheral blood mononuclear cell
ROS: Reactive oxygen species
SOD: Superoxide dismutase
Sp1: Specificity protein 1
Sp3: Specificity protein 3
TB: Tuberculosis
z-VAD: Z-VAD-fmk.

## Supplementary Materials

Supplementary materials Table S1: primers used. Figure S1: the effect of JFD on the viability of THP-1 cells, H37Ra growth, and phagocytosis. Figure S2: the effects of JFD on the secretion of TNF*α*, IL-1*β*, and autophagy. Figure S3: the effects of JFD on the activation of AKT-mTOR and NF-*κ*B signaling pathway. Figure S4: the effects of JFD on the expression of SOD1 and SOD3. Figure S5: the effects of JFD on the alkylation of cysteine residues on Keap1. Figure S6: pharmacokinetic analysis of JFD *in vivo*.
