# Supplementary material for: JFD, a Novel Natural Inhibitor of Keap1 Alkylation, Suppresses Intracellular Mycobacterium Tuberculosis Growth through Keap1/Nrf2/SOD2-Mediated ROS Accumulation
Source: Oxid Med Cell Longev. 2023 Feb 10;2023:6726654. doi: 10.1155/2023/6726654 (PMC9937762; doi:10.1155/2023/6726654)
Supplement: Supplementary materials — Table S1: primers used. Figure S1: the effect of JFD on the viability of THP-1 cells, H37Ra growth, and phagocytosis. Figure S2: the effects of JFD on the secretion of TNFα, IL-1β, and autophagy. Figure S3: the effects of JFD on the activation of AKT-mTOR and NF-κB signaling pathway. Figure S4: the effects of JFD on the expression of SOD1 and SOD3. Figure S5: the effects of JFD on the alkylation of cysteine residues on Keap1. Figure S6: pharmacokinetic analysis of JFD in vivo. [file 6726654.f1.pdf]

Oxidative Medicine and Cellular Longevity

JFD, a Novel Natural Inhibitor of Keap1 Alkylation, Suppresses Intracellular *Mycobacterium tuberculosis* Growth through Keap1/Nrf2/SOD2-Mediated ROS Accumulation

Haoqiang Wan,[1,2] Yi Cai,[3] Lingyun Xiao,[1] Yunzhi Ling,[1] Lanlan Ge,[1,2] Siwei Mo,[3] Qiujie Xie,[1] Shusong Peng,[1] Boping Zhou,[1] Xiaobin Zeng,[1,2,3] and Xinchun Chen[3]

[1]*Center Lab of Longhua Branch and Department of Infectious Disease, Shenzhen People's Hospital (The Second Clinical Medical College, Jinan University; The First Affiliated Hospital, Southern University of Science and Technology), Shenzhen 518020, Guangdong Province, China*

[2]*Department of pathology (Longhua Branch), Shenzhen People's Hospital, 2nd Clinical Medical College of Jinan University, Shenzhen 518020, Guangdong Province, China*

[3]*Guangdong Key Laboratory of Regional Immunity and Diseases, Department of Pathogen Biology, Shenzhen University School of Medicine, Shenzhen 518120, Guangdong Province, China*

*Correspondence should be addressed to Boping Zhou, zhoubp@hotmail.com; Xiaobin Zeng, zeng.xiaobin@szhospital.com; and Xinchun Chen, chenxinchun@szu.edu.cn

Table S1. Primers used for real-time PCR experiments.

**SOD1-F**	**5’-GGTGGGCCAAAGGATGAAGAG-3’**
**SOD1-R**	**5’-CCACAAGCCAAACGACTTCC-3’**
**SOD2-F**	**5’-GGAAGCCATCAAACGTGACTT-3’**
**SOD2-R**	**5’-CCCGTTCCTTATTGAAACCAAGC-3’**
**SOD3-F**	**5’-CATCCACGTGCACCAGTTCG-3’**
**SOD3-R**	**5’-TACCTCCAGAGGCTGCCGTC-3’**
**Nrf2-F**	**5’-TCAGCGACGGAAAGAGTATGA-3’**
**Nrf2-R**	**5’-CCACTGGTTTCTGACTGGATGT-3’**
**Foxo3a-F**	**5’-TCACGCACCAATTCTAACGC-3’**
**Foxo3a-R**	**5’-CACGGCTTGCTTACTGAAGG-3’**
**Sp1-F**	**5’-TGGCAGCAGTACCAATGGC-3’**
**Sp1-R**	**5’-CCAGGTAGTCCTGTCAGAACTT-3’**
**Sp3-F**	**5’-AGTGGGCAGTATGTTCTTCCC-3’**
**Sp3-R**	**5’-GACTGGATCTGTGGTATCACTTG-3’**
**GAPDH-F**	**5’-AGAAGGCTGGGGCTCATTTG-3’**
**GAPDH-R**	**5’-AGGGGCCATCCACAGTCTTC-3’**

Supplementary Figure Captions

Figure S1. (A) The effect of different concentrations of JFD on the viability of THP-1 cells, **(B)** H37Ra growth in 7H9 medium and **(C)** phagocytosis of H37Ra by THP-1 cells. JFD, japoflavone D; M. tuberculosis, Mycobacterium tuberculosis; MFI, mean fluorescence intensity. Graphs show the mean ± SD of triplicate wells and are representative of three independent experiments.

Figure S2. The effects of JFD treatment for 24 hours on the secretion of TNFα **(A)**, IL-1β **(B)** and autophagy **(C)** in H37Ra infected THP-1 cells. **(D)** The effects of CHQ and 3MA treatment on the anti-tuberculosis activity of JFD. JFD, japoflavone D; CFU, clone forming units; CHQ, chloroquine; 3MA, 3-methyladenine. Graphs show mean ± SD of triplicate wells and represent three independent experiments.

Figure S3. The effects of JFD treatment for 24 hours on the activation of AKT/mTOR and NF-κB signaling pathway in H37Ra infected THP-1 cells. JFD. JFD, japoflavone D.

Figure S4. The effects of JFD treatment for 24 hours on the expression of SOD1 **(A)** and SOD3 **(B)** in H37Ra infected THP-1 cells. JFD, japoflavone D. Graphs show mean ± SD of triplicate wells and represent three independent experiments.

Figure S5. The effects of JFD on the alkylation of cysteines residues on Keap1. **(A-D)** Keap1 was incubated with CDDO-EA for 2h at room temperature.

Representative MS/MS secondary spectrums of the peptide containing the CDDO-EA modified Cys14, Cys257 and Cys319. (E-H) Keap1 was incubated with CDDO-EA in the presence of JFD for 2h at room temperature. Representative MS/MS secondary spectrums of the peptide containing the carbamidomethylated Cys14, Cys257 and Cys319. JFD, japoflavone D; Cys, cysteine.

Figure S1

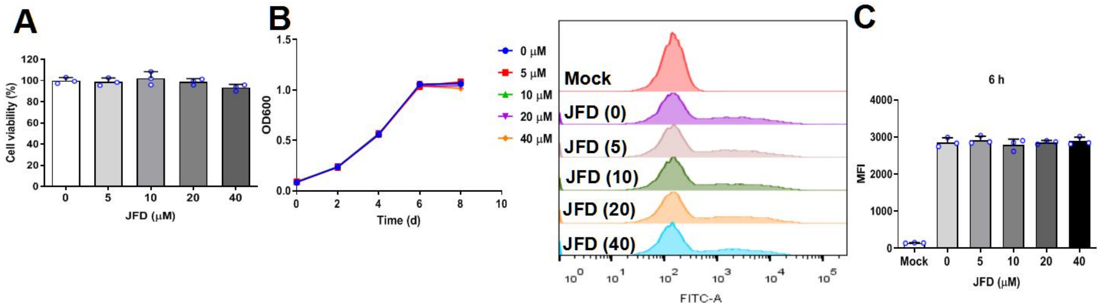
A
Cell viability (%)
0
20
40
60
80
100
120
0
5
10
20
40
JFD (μM)
B
OD600
0.0
0.5
1.0
1.5
0
2
4
6
8
10
Time (d)
0 μM
5 μM
10 μM
20 μM
40 μM
Mock
JFD (0)
JFD (5)
JFD (10)
JFD (20)
JFD (40)
FITC-A
C
6 h
MFI
0
1000
2000
3000
4000
Mock
0
5
10
20
40
JFD (μM)

Figure S2

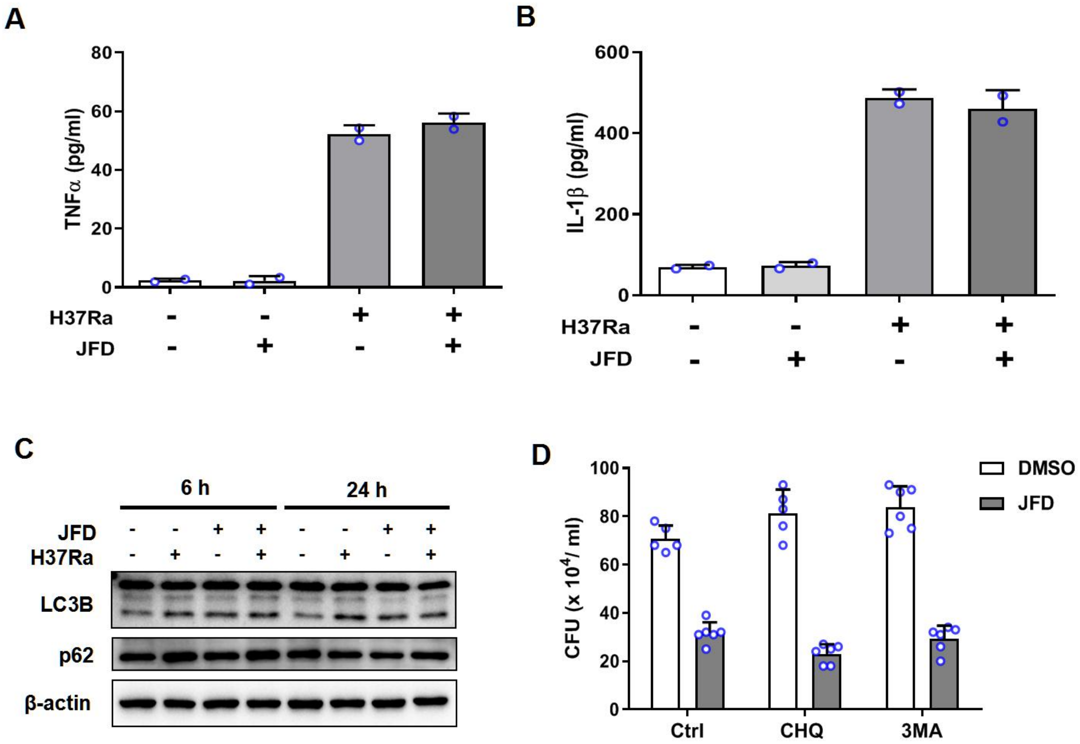
A
TNFα (pg/ml)
80
60
40
20
0
H37Ra - - + +
JFD - + - +
B
IL-1β (pg/ml)
600
400
200
0
H37Ra - - + +
JFD - + - +
C
6 h
24 h
JFD - - + + - - + +
H37Ra - + - + - + - +
LC3B
p62
β-actin
D
CFU (x 10⁴/ ml)
100
80
60
40
20
0
Ctrl
CHQ
3MA
DMSO
JFD

Figure S3

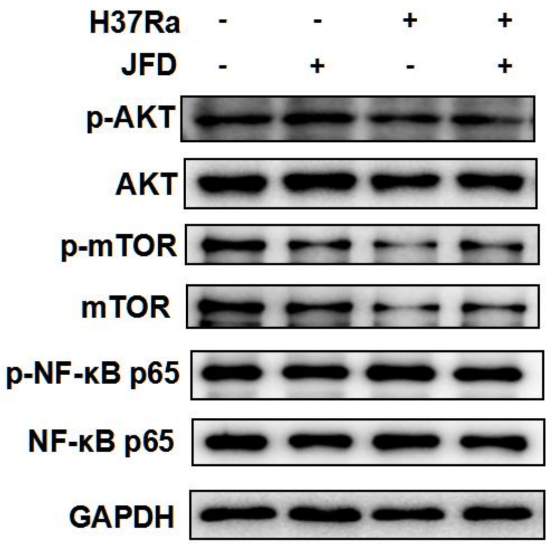

Figure S4

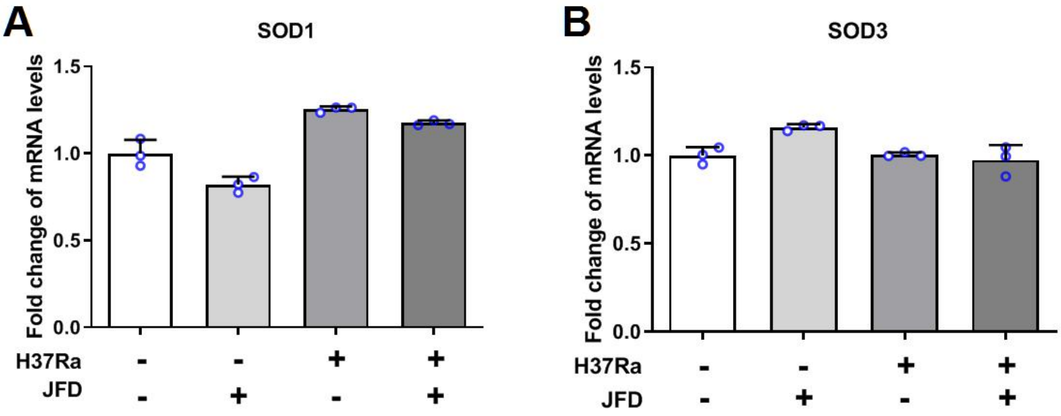

Figure S5

A

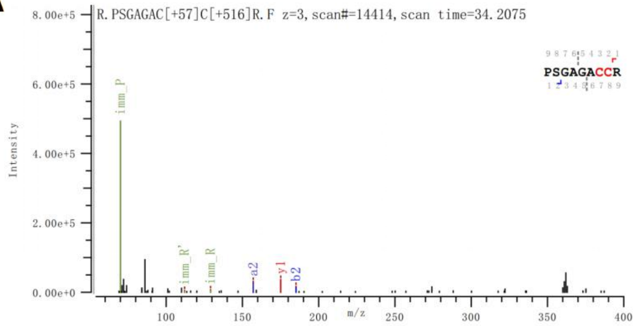

2563: PSGAGACCR								
#	a calc.	b calc.	b-18 calc.	b++ calc.	Seq.	y calc.	y++ calc.	#
1	70.0651	98.0600	80.0495	49.5337	P			9
2	157.0972	185.0921	167.0815	93.0497	S	1297.6479	649.3276	8
3	214.1186	242.1135	224.1030	121.5604	G	1210.6159	605.8116	7
4	285.1557	313.1506	295.1401	157.0790	A	1153.5945	577.3009	6
5	342.1772	370.1721	352.1615	185.5897	G	1082.5573	541.7823	5
6	413.2143	441.2092	423.1986	221.1082	A	1025.5359	513.2716	4
7	573.2449	601.2398	583.2293	301.1236	C	954.4988	477.7530	3
8	1192.5941	1220.5890	1202.5785	610.7982	C	794.4681	397.7377	2
9					R	175.1190	88.0631	1

B

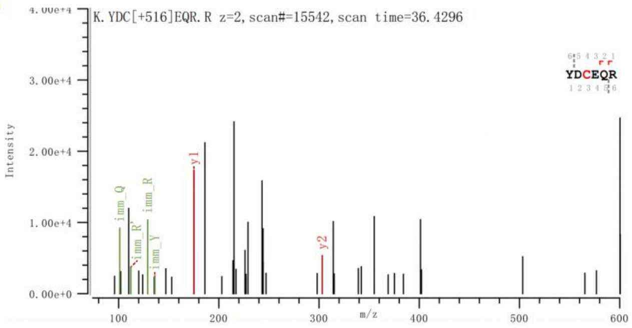

2296: YDCEQR							
#	a calc.	b calc.	b-18 calc.	Seq.	y calc.	y++ calc.	#
1	136.0757	164.0706	146.0600	Y			6
2	251.1026	279.0975	261.0870	D	1166.5962	583.8018	5
3	870.4518	898.4467	880.4362	C	1051.5693	526.2883	4
4	999.4944	1027.4893	1009.4788	E	432.2201	216.6137	3
5	1127.5530	1155.5479	1137.5373	Q	303.1775	152.0924	2
6				R	175.1190	88.0631	1

C

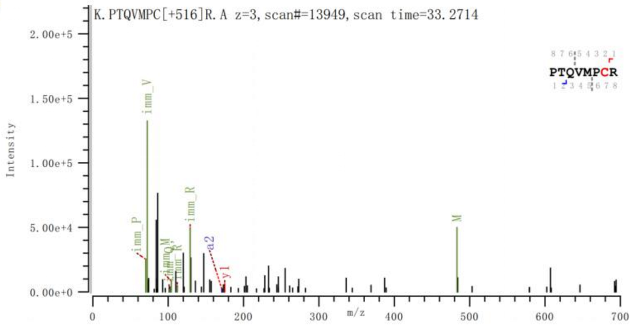

3146: PTQVMPCR								
#	a calc.	b calc.	b-18 calc.	b++ calc.	Seq.	y calc.	y++ calc.	#
1	70.0651	98.0600	80.0495	49.5337	P			8
2	171.1128	199.1077	181.0972	100.0575	T	1350.7360	675.8717	7
3	299.1714	327.1663	309.1557	164.0868	Q	1249.6884	625.3478	6
4	398.2398	426.2347	408.2241	213.6210	V	1121.6298	561.3185	5
5	529.2803	557.2752	539.2646	279.1412	M	1022.5614	511.7843	4
6	626.3330	654.3279	636.3174	327.6676	P	891.5209	446.2641	3
7	1245.6822	1273.6771	1255.6666	637.3422	C	794.4681	397.7377	2
8					R	175.1190	88.0631	1

Figure S5 (continued)

D

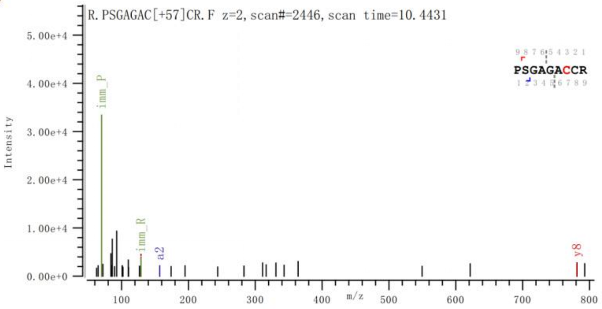

408: PSGAGACCR

#	a calc.	b calc.	b-18 calc.	Seq.	y calc.	y++ calc.	#
1	70.0651	98.0600	80.0495	P			9
2	**157.0972**	185.0921	167.0815	S	**781.3079**	391.1576	8
3	214.1186	242.1135	224.1030	G	694.2759	347.6416	7
4	285.1557	313.1506	295.1401	A	637.2545	319.1309	6
5	342.1772	370.1721	352.1615	G	566.2173	283.6123	5
6	413.2143	441.2092	423.1986	A	509.1959	255.1016	4
7	573.2449	601.2398	583.2293	C	438.1588	219.5830	3
8	676.2541	704.2490	686.2385	C	278.1281	139.5677	2
9				R	175.1190	88.0631	1

E

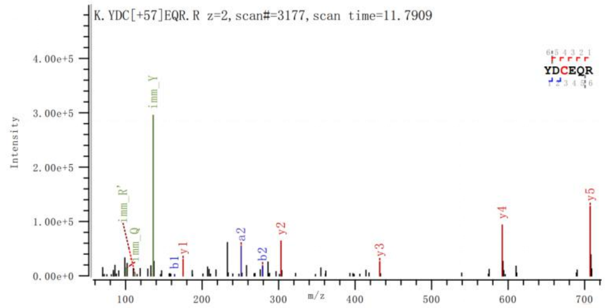

417: YDCEQR

#	a calc.	b calc.	b-18 calc.	Seq.	y calc.	y++ calc.	#
1	136.0757	**164.0706**	146.0600	Y			6
2	**251.1026**	**279.0975**	261.0870	D	**707.2777**	354.1425	5
3	411.1333	439.1282	421.1176	C	**592.2508**	296.6290	4
4	540.1759	568.1708	550.1602	E	**432.2201**	216.6137	3
5	668.2344	696.2294	678.2188	Q	**303.1775**	152.0924	2
6				R	**175.1190**	88.0631	1

F

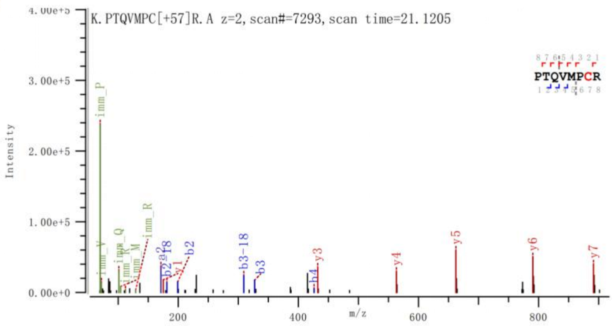

522: PTQVMPCR

#	a calc.	b calc.	b-18 calc.	Seq.	y calc.	y++ calc.	#
1	70.0651	98.0600	80.0495	P			8
2	**171.1128**	**199.1077**	**181.0972**	T	**891.4175**	446.2124	7
3	299.1714	**327.1663**	**309.1557**	Q	**790.3698**	395.6885	6
4	398.2398	**426.2347**	408.2241	V	**662.3112**	331.6593	5
5	529.2803	557.2752	539.2646	M	**563.2428**	282.1251	4
6	626.3330	654.3279	636.3174	P	**432.2024**	216.6048	3
7	786.3637	814.3586	796.3480	C	335.1496	168.0784	2
8				R	**175.1190**	88.0631	1

Raw of Western Blot

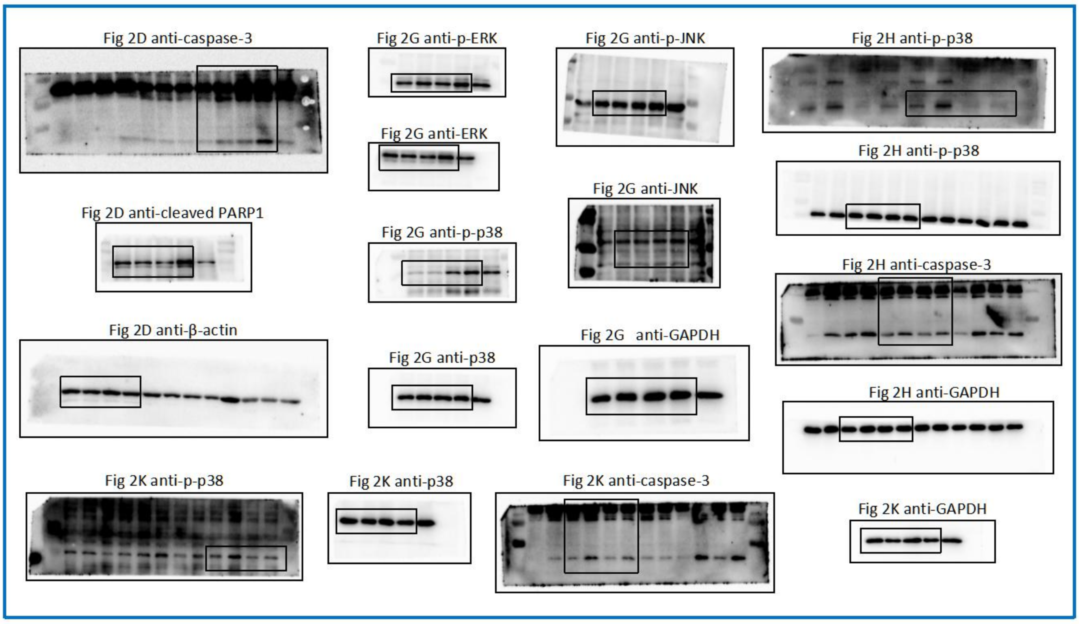

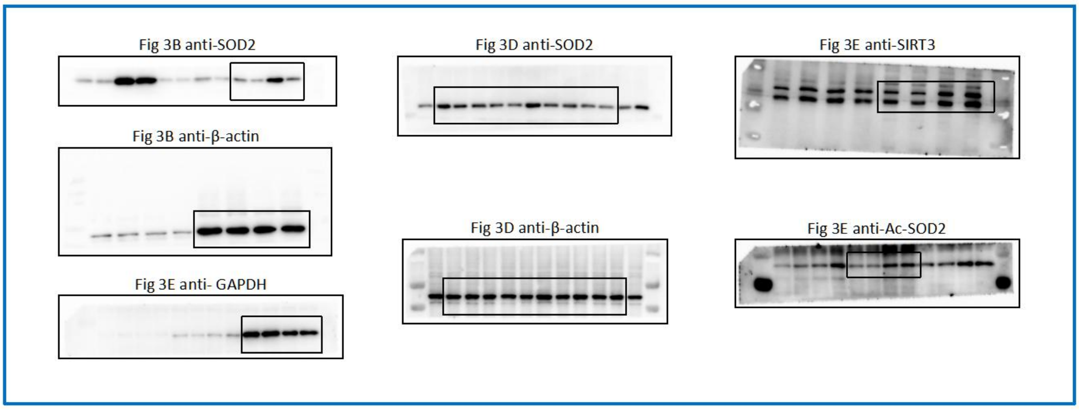

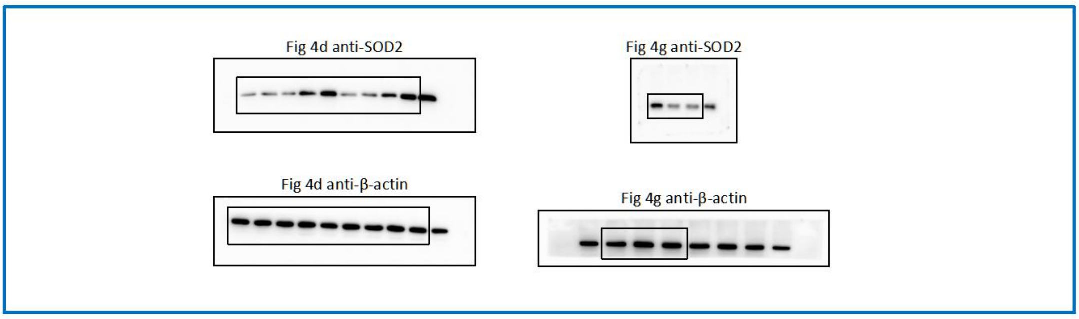

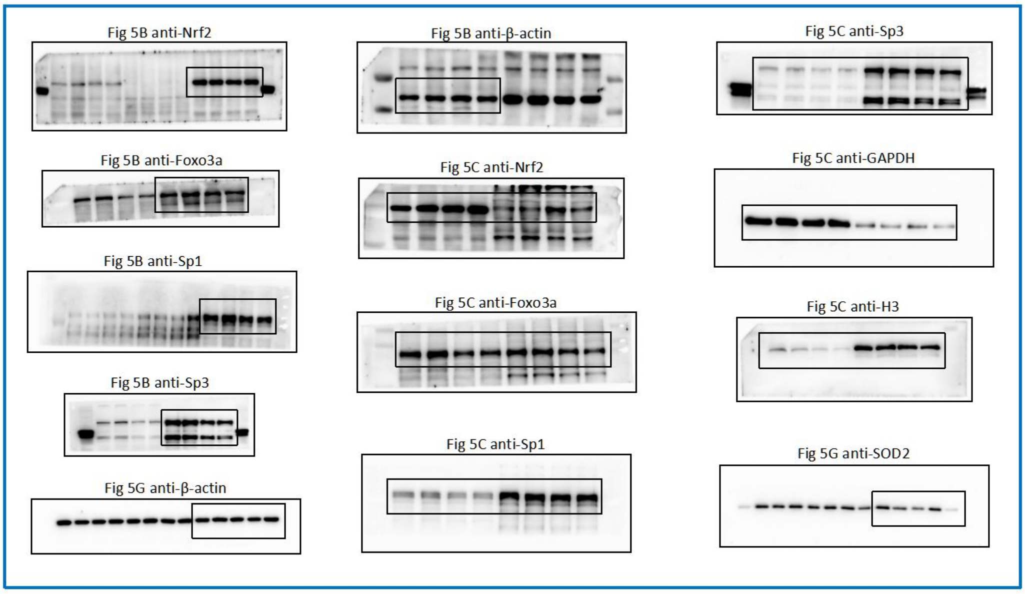
Fig 5B anti-Nrf2
Fig 5B anti-β-actin
Fig 5C anti-Sp3
Fig 5B anti-Foxo3a
Fig 5C anti-Nrf2
Fig 5C anti-GAPDH
Fig 5B anti-Sp1
Fig 5C anti-Foxo3a
Fig 5C anti-H3
Fig 5B anti-Sp3
Fig 5C anti-Sp1
Fig 5G anti-SOD2
Fig 5G anti-β-actin

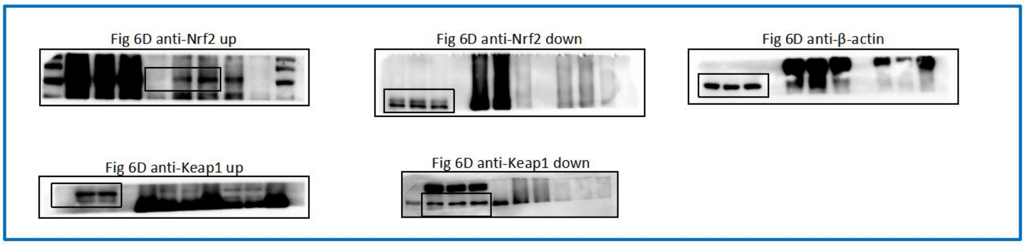
Fig 6D anti-Nrf2 up
Fig 6D anti-Nrf2 down
Fig 6D anti-β-actin
Fig 6D anti-Keap1 up
Fig 6D anti-Keap1 down

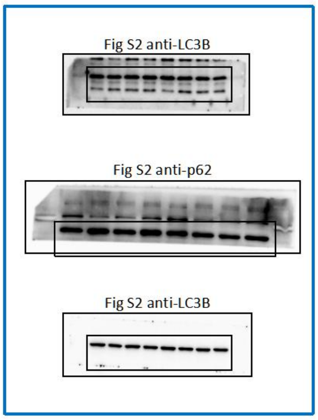
Fig S2 anti-LC3B
Fig S2 anti-p62
Fig S2 anti-LC3B

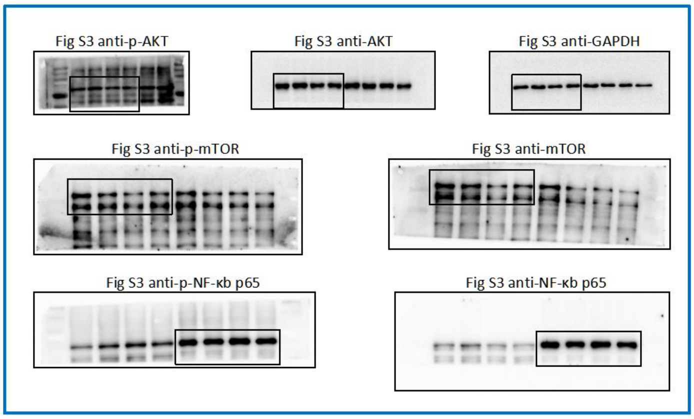
Fig S3 anti-p-AKT
Fig S3 anti-AKT
Fig S3 anti-GAPDH
Fig S3 anti-p-mTOR
Fig S3 anti-mTOR
Fig S3 anti-p-NF-κb p65
Fig S3 anti-NF-κb p65
